# Influence of Drying Protocol on the Setting and Crystalline Phase Formation of Calcium Silicate‐Based Sealers

**DOI:** 10.1111/aej.70044

**Published:** 2025-12-09

**Authors:** Thiago Bessa Marconato Antunes, Ana Cristina Padilha Janini, Isis Hinnebusch, Nilvan Alves da Silva, Gaspar Darin Filho, Elda Xavier Souza Silveira, Mário Alexandre Coelho Sinhoreti, Bruno Martini Guimarães, Marina Angélica Marciano

**Affiliations:** ^1^ Department of Restorative Dentistry, Faculdade de Odontologia de Piracicaba (FOP), Universidade Estadual de Campinas (UNICAMP), Piracicaba São Paulo Brazil; ^2^ Institute of Chemistry, Universidade Estadual de Campinas (UNICAMP) Campinas Brazil; ^3^ Department of Mining and Petroleum Engineering, Escola Politécnica Universidade de São Paulo (USP) São Paulo Brazil; ^4^ Department of Product and Process, Faculdade de Engenharia Elétrica e de Computação (FEEQ). Universidade Estadual de Campinas (UNICAMP), Campinas São Paulo Brazil; ^5^ Dental School Universidade Federal De Alfenas Alfenas Minas Gerais Brazil

**Keywords:** calcium silicate, FT‐IR, Raman spectroscopy, root canal filling material, X‐ray diffraction analysis

## Abstract

The objective of this study was to assess the hydration, crystalline structure, and setting of bioceramic ready‐to‐use and powder/liquid sealers in dry and wet root canal dentine. Thirty‐two single‐rooted human teeth were embedded in acrylic resin and sectioned into 2‐mm segments. Canals were filled under dry or moist conditions using BioRoot RCS or Bio‐C Sealer. Setting time was assessed using a modified Gilmore needle. Hydration was analyzed via Raman and FT‐IR spectroscopy and X‐ray diffraction (XRD) after 28 days. Data were analysed using two‐way ANOVA and Tukey test (*α* = 5%). Bio‐C Sealer had shorter setting times in moist canals (*p* < 0.01), while BioRoot showed no significant difference (*p* > 0.05). Raman identified a calcite peak (1150 cm^−1^), and FT‐IR showed water reduction over time. XRD detected calcium hydroxide in moist Bio‐C Sealer and apatite/calcite in BioRoot. Dentine moisture is crucial for the setting of ready‐to‐use calcium silicate‐based sealers.

## 1 Introduction

Root canal filling is a crucial phase in endodontic treatment and is of great importance for overall success [1]. The gutta‐percha, combined with a sealer, must fill the spaces previously occupied by the dental pulp and promote a homogeneous sealing along the entire dimension of the canals, preventing bacterial colonisation [2]. Calcium silicate‐based sealers exhibit biological properties, including tissue biocompatibility [3], bioactivity [4], the ability to differentiate osteoblasts [5] and antimicrobial potential attributed to increased pH [6].

Calcium silicate‐based sealers are available in two main commercial forms: powder/liquid and ready‐to‐use. Their bioactivity depends on the production of calcium hydroxide through the hydration reaction [7, 8, 9]. Powder/liquid sealers begin setting immediately upon mixing tricalcium and dicalcium silicate with water [4]. In contrast, ready‐to‐use sealers, available in syringe form, contain tricalcium and dicalcium silicate combined with a dispersing agent (polymer) but lack water, requiring external moisture to initiate setting [7, 10, 11]. Consequently, these sealers rely on the moisture present in dentine to complete the setting process [11, 12].

Previous studies have evaluated different drying protocols for root canals before filling with hydrophilic sealers [13, 14, 15]. Evidence suggests that these materials achieve higher bond strength and better performance when dentine remains slightly moist prior to filling [15]. Conversely, excessive drying can hinder their penetration into dentinal tubules [14]. In general, it has been reported that extremes, either excessive moisture or complete dryness, are detrimental to excellent sealing [15].

Despite the advancements in calcium silicate‐based sealers, uncertainties remain regarding their setting reactions within root canals. Studies on drying protocols are needed to assess whether the moisture in dentine is indeed sufficient to encompass the entire sealer mass and react with the calcium silicate particles [15]. The objective of this study was to evaluate the hydration of ready‐to‐use sealers compared to sealers presented in powder/liquid form, filled in dry and wet human root canal teeth, at 1 and 28 days after filling.

Therefore, the null hypothesis tested was that there would be no significant differences in setting time, hydration or crystalline phase formation between calcium silicate‐based sealers placed in dry and moist root canal dentine.

## 2 Materials and Methods

**FIGURE 1:**
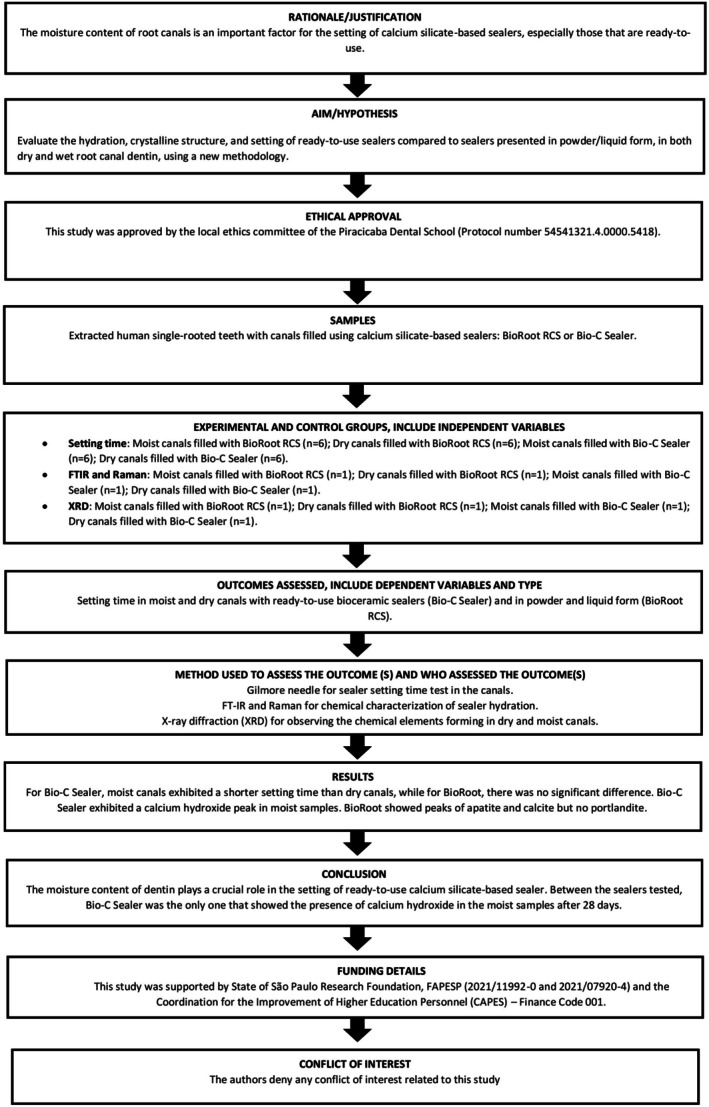
Flowchart according to the PRILE 2021 guidelines.

The manuscript of this laboratory study has been written according to the Preferred Reporting Items for Laboratory Studies in Endodontology (PRILE) 2021 guidelines (Figure 1) [16].

### 2.1 Preparation of the Materials

The sample size was determined using G*Power 3.1 software (Heinrich Heine, University Dusseldorf) based on prior studies (Nagas et al. 2023 [13]) and (Pelozo et al. 2023, [15]). A total of six groups were formed, with an estimated standard deviation of 0.44 and a minimum detectable difference of 1.67, yielding a sample size of 6 teeth per group (*n* = 6).

Thirty‐two human teeth with straight root canals and closed apices were selected. The teeth were sectioned at the cemento‐enamel junction using a cutting machine (ISOMET 1000; Buehler, Lake Bluff, IL, USA) equipped with a 0.3‐mm diamond disc. Root canals were instrumented with a #80 K‐file (Dentsply Maillefer, Tulsa, USA) and irrigated with 2.5% sodium hypochlorite for decontamination.

A Largo Peeso #6 drill (Dentsply Maillefer, Tulsa, USA) was used to enlarge the canal to a uniform diameter of 2 mm. A digital calliper (500–463; Mitutoyo Corporation, Kanagawa, Japan) confirmed the length. Passive ultrasonic irrigation (PUI) was performed using a 20/0.01 E1‐Irrisonic insert (Helse ultrasonic, Santa Rosa de Viterbo, SP, Brazil) coupled to an ultrasonic device (Newtron Booster, Mount Laurel, USA). The protocol involved three cycles of 20 s with 2.5% NaOCl, 17% EDTA and NaOCl again. After irrigation, the samples were rinsed with 6 mL distilled water and divided into two groups based on the endodontic sealer (Table 1):

- Ready‐to‐use: Bio‐C Sealer (Angelus, Londrina, PR, Brazil)
- Powder/liquid: BioRoot RCS (Septodont, Saint‐Maur‐des‐Fosses, France)

**TABLE 1:** Composition of root canal sealers and their batch number.

Material	Batch	Composition
BioRoot RCS (Septodont)	Powder B29203 Liquid B29182	Tricalcium silicate, zirconium oxide, water. Calcium chloride, water‐soluble polymer (polycarboxylate)
Bio‐C Sealer (Angelus)	60 406	Calcium silicate, calcium aluminate, calcium oxide, zirconium oxide, iron oxide. Silicon dioxide and polyethylene glycol

### 2.2 Sample Preparation

**FIGURE 2:**
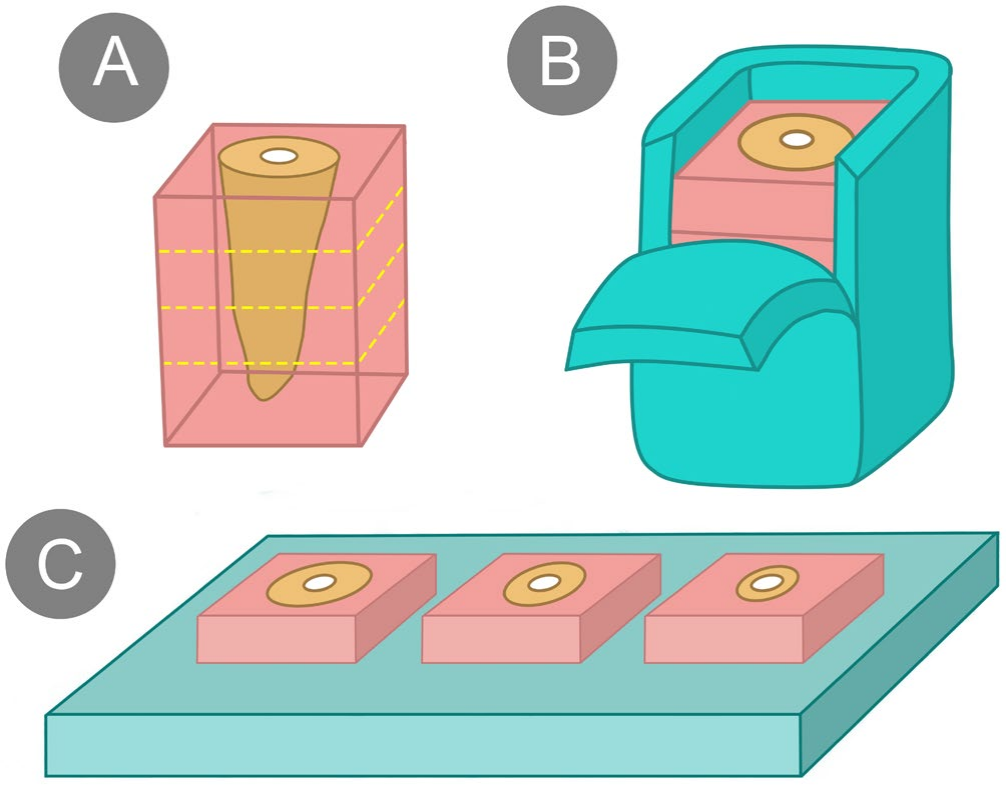
Root filled with BioRoot or Bio‐C Sealer cement embedded in an acrylic resin block. After embedding, the root was sectioned into three slices of 2 mm corresponding to the cervical, middle and apical thirds (A). The sectioned resin block was placed on a silicone pad, and on one side, a cut was made to open and close for the removal of slices from each third (B). To assess the setting time, each slice was placed on a glass plate for the positioning of the Gilmore needle on the cement surface (C). After the test, the slices were returned to the silicone pad.

The teeth were embedded in acrylic resin‐filled square containers (100 mm^2^ base, 30 mm height). Once polymerised, roots were sectioned into cervical, middle and apical thirds (2 mm thick). Condensation silicone (Zetaplus Putty, Zhermack SpA, Badia Polesine, Rovigo, Italy) moulds supported the samples to maintain moisture (Figure 2).

Each sealer group was subdivided into dry (*n* = 6) and wet (*n* = 6) canals, following Pelozo et al. 2023, [15]:

- **Dry**: The canals were dried using a 2‐mm‐diameter suction tip (White Mac tip; Ultradent, Indaiatuba, SP, Brazil) for 5 s at the orifices of the root canals, followed by aspiration with a 0.48 mm capillary tip (Ultradent) for 5 s. Next, 80 paper points (Dentsply Maillefer, Ballaigues, Switzerland) were inserted to the working length until completely dry. The dryness of the final paper point was confirmed visually.
- **Moist**: Root canal drying was performed with the White Mac tip, followed by capillary tip aspiration according to the previous protocol, without using paper points. Following the grouping, the sealers were individually placed into the corresponding canals without the use of gutta‐percha.

Sealers were applied without gutta‐percha. Bio‐C Sealer was injected with an applicator tip, while BioRoot RCS was mixed (powder with five drops of liquid for 1 min) and inserted using a #80 gutta‐percha cone. An endodontic condenser (Golgran, São Caetano do Sul, SP, Brazil) ensured uniform distribution. Samples were stored at 37°C with 95% humidity.

### 2.3 Setting Time

Samples (*n* = 12) were randomised and subdivided into BioRoot (*n* = 6) and Bio‐C Sealer (*n* = 6). Based on ISO 6876/2012 and Silva et al. (2021) [17], setting time was assessed using a 50 g Gilmore needle (Odeme Dental Research; Luzerna, SC, Brazil) with a 1 mm tip. The needle was applied every 10 min until no indentation was observed, indicating complete setting.

### 2.4 Raman Spectroscopy

Raman spectroscopy analysed chemical functional groups at three time points: fresh, 1 day and 28 days. One sample from each subgroup (dry and wet) per sealer was used. Spectra were obtained using a 785 nm laser (Cobolt 08 series 0785 08‐11‐0500‐200; Hubner Photonics, Kassel, Germany) at 21 mW, coupled to a spectrometer (Andor/Oxford SR‐500i C‐SIL; Shamrock, Belfast, UK) with a CCD camera (Andor/Oxford iDUS 416 DU416A‐LDC‐DD; Shamrock) at −65°C. Real‐time Raman spectra were obtained by using an adjustable laboratory‐made spectrometer. Spectral resolution ranged from 100 to 2000 cm^−1^. Data processing included Weighted Least Squares baseline correction and Savitzky–Golay smoothing.

### 2.5 Fourier‐Transform Infrared Spectroscopy (FT‐IR)

FT‐IR evaluated chemical functional groups at fresh, 1 day and 28 days. One sample from each subgroup (dry and wet) per sealer was analysed using the potassium bromide (KBr) pellet method. KBr pellets were prepared by mixing 0.05 g of sealer with 0.1 g KBr, ground and pressed under vacuum (RIIK 10‐tonne ring press) for 3 min. Spectra were recorded with a calibrated FT‐IR spectrometer (Vertex 70v; Bruker, Billerica, MA, USA) in transmittance mode, at 4 cm^−1^ resolution, from 400 to 4000 cm^−1^.

### 2.6 X‐Ray Diffraction (XRD)

XRD was conducted on single samples from the middle third of each sealer group (dry and wet). Samples were stored in a vacuum desiccator for 28 days. One tooth was used for each subgroup (dry and wet) for both Bio‐C and BioRoot. The sealer in the same tooth was evaluated after 1 and 28 days. The crystalline phases were identified using an X‐ray diffractometer (PANalytical Empyrean, Almelo, Netherlands) with CuKa radiation (45 kV, 40 mA). Scans ranged from 5° to 70° 2θ, with a 0.02° step size. Crystalline compounds were identified using the FIZ Karlsruhe's Inorganic Crystal Structure Database (ICSD) with codes: 98–006‐7247 (Zirconia‐ZrO_2_), 98–001‐2748 (Hatrurite‐Ca_3_SiO_5_), 98–004‐7697 (Baddeleyite‐ZrO_2_) and 98–004‐6326 (Portlandite‐Ca(OH)_2_).

Raman and FT‐IR data were processed using MATLAB and Origin software, while Highscore Plus 5.0 analysed XRD data.

### 2.7 Statistical Analysis

JASP software (version 0.9.2; University of Amsterdam, Netherlands) was used for two‐way ANOVA to analyse within‐ and between‐group effects. Tukey post hoc tests assessed differences at a 5% significance level (*p* < 0.05).

## 3 Results

### 3.1 Assessment of Setting Time

The setting time (hours) of wet and dry root canal dentine is shown in Table 2. Regarding the setting time, the Bio‐C Sealer showed a statistically significant difference between wet and dry canal dentine within the same thirds (*p* < 0.01). Nevertheless, when considering the thirds within each subgroup (dry and wet), no statistical difference was observed (*p* > 0.05). In contrast, for the BioRoot sealer, there was no statistical difference in setting time between wet and dry canal dentine within the same thirds (*p* > 0.05). Additionally, there was no statistical difference between the thirds of each subgroup associated with this sealer (*p* > 0.05).

**TABLE 2:** Mean and standard deviation of setting time (h) for Bio‐C Sealer and BioRoot sealers in the cervical (CT), middle (MT) and apical thirds (AT).

Setting time (h)				
	Bio‐C Sealer		BioRoot RCS	
Third	Dry	Moist	Dry	Moist
CT	47.45 ± 4.52 ^Aa^	16.19 ± 8.97 ^Ba^	7.14 ± 2.25 ^Aa^	9.48 ± 0.47 ^Aa^
MT	52.23 ± 3.8 ^Aa^	28.03 ± 8.8 ^Ba^	10.05 ± 1.97 ^Aa^	9.81 ± 0.51 ^Aa^
AT	51.71 ± 3.71 ^Aa^	25.65 ± 8.62 ^Ba^	10.00 ± 2.02 ^Aa^	9.80 ± 0.51 ^Aa^

*Note:* Different uppercase letters indicate statistical differences within the same sealer group for wet and dry canals. Similar lowercase letters indicate no statistical difference between the thirds within each treatment subgroup (wet and dry) in the same sealer group.

### 3.2 Assessment of Spectroscopy

**FIGURE 3:**
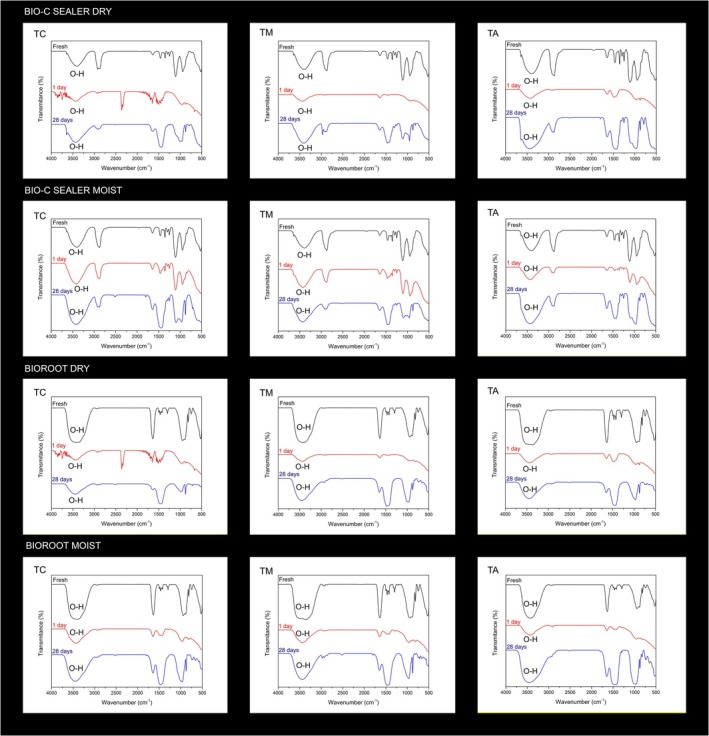
FT‐IR spectra of Bio‐C Sealer and BioRoot filled in dry and wet root canals. Water peaks are evident at 3400 cm^−1^.

**FIGURE 4:**
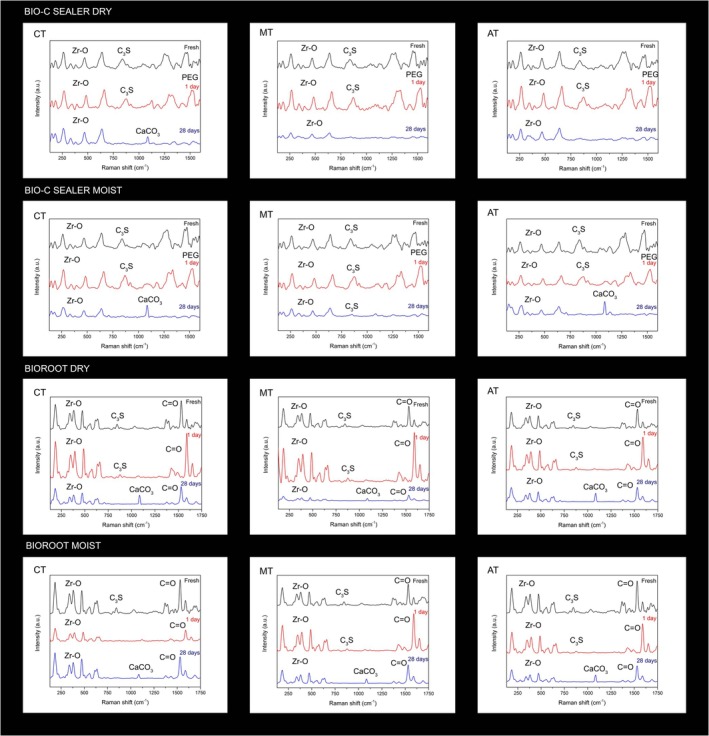
Raman spectra of the Bio‐C Sealer and BioRoot groups filled in dry and wet root canals. Peaks associated with zirconium oxide are detected between 178 and 639 cm^−1^. Calcite (CaCO_3_) peaks are observed at 1150 cm^−1^. Peaks corresponding to the C=O bond, indicative of the polycarboxylate polymer chain, are observed between 1400 and 1600 cm^−1^. In the spectral range near 1500 cm^−1^, peaks representing the polyethylene glycol (PEG) polymer chain are identified.

Figures 3 and 4, respectively, represent FT‐IR and Raman spectroscopy outcomes. The FT‐IR spectra of the Bio‐C Sealer exhibit prominent water peaks on the first day when the wet root canal dentine was moist, contrasting with the spectra of the dry Bio‐C Sealer during the same period at 3400 cm^−1^. After 28 days, the water peaks become highly noticeable. In the fresh form, the dry samples displayed a peak of the OH bond at 3400 cm^−1^. The FT‐IR spectra of both moist and dry root canals for BioRoot reveal prominent peaks in their fresh forms, with intensities surpassing those of the Bio‐C Sealer. Within a day, the peaks in both moist and dry root canal groups are evident; however, in the moist root canal group, the peaks are more pronounced compared to the dry group. Similar to the Bio‐C group, the water peak increased after 28 days. Both the dry samples of Bio‐C Sealer and those of BioRoot exhibited peaks at 2300 cm^−1^ in the cervical third 1 day after filling related to carbon dioxide.

Prolonged peaks of the C‐OH bond were observed at 1500 cm^−1^, more pronounced at 28 days for both BioRoot and Bio‐C Sealer. At 1150 cm^−1^, a Raman peak was observed in the cervical third after 28 days for the dry root canal for the Bio‐C Sealer group and in the middle and apical thirds for the moist root canal for the Bio‐C Sealer group during the same period. The same peak was observed in both the moist and dry root canal for the BioRoot groups in all thirds, with a more pronounced presence in the apical third. This peak is potentially associated with calcite (CaCO_3_).

### 3.3 X‐Ray Diffraction

**FIGURE 5:**
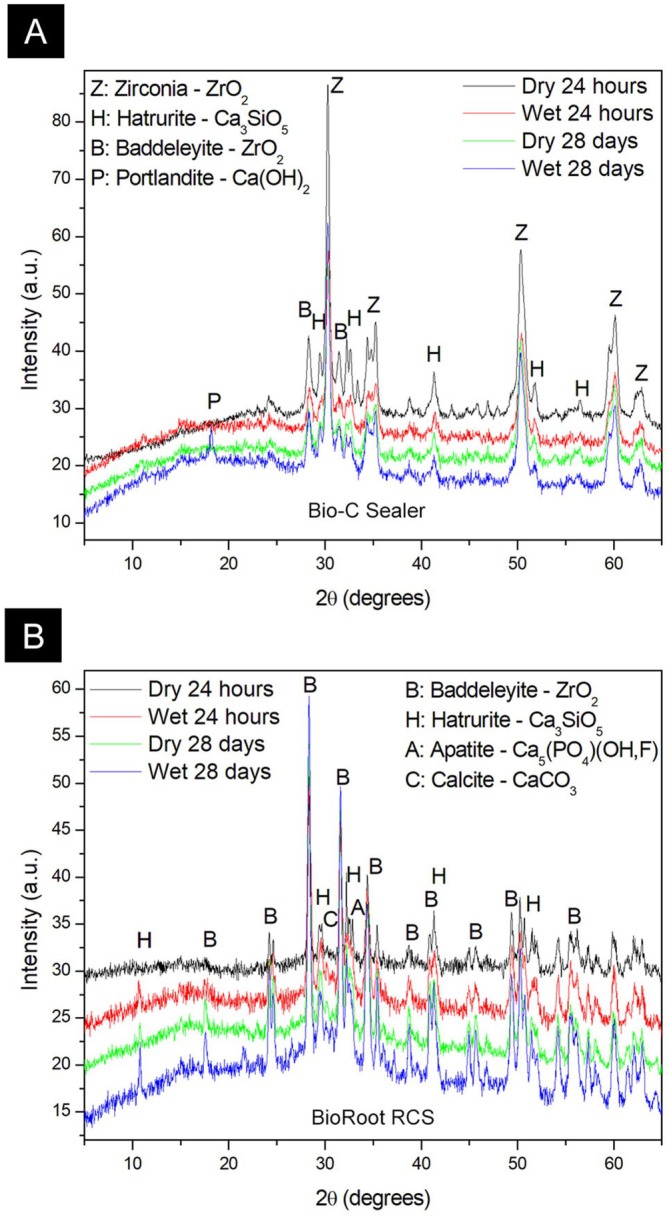
Figure 5A reveals the following: The diffractograms for Sample Bio‐C showing mainly zirconium oxide ZrO2 (Z, B) and tricalcium silicate Ca3SiO5 (H). A low content of calcium hydroxide Ca(OH)2 (P) was identified only for wet 28 days. Amorphous material is evidenced by the existence of a characteristic broad band between 15 and 40 degrees and was observed in all diffractograms of this batch of samples. Figure 5B reveals the following: (a) in contrast to Sample Bio‐C, the diffractograms for Sample BioRoot detected only the phase B of zirconium oxide besides tricalcium silicate (H). In addition, low evidences of calcium carbonate (calcite) and calcium phosphate (apatite) were observed. Amorphous material was not detected significantly exclusively for the sample Dry 24 h. All the other samples' amorphous phase was identified.

Figure 5 represents XRD patterns in the 5°–70° 2θ range and phase analysis. The diffractograms for Bio‐C Sealer (Figure 5A) showed mainly zirconium oxide ZrO_2_ and tricalcium silicate Ca_3_SiO_5_. A low content of calcium hydroxide Ca(OH)_2_ was identified only for the wet canal group after 28 days. Amorphous material is evidenced by the existence of a characteristic broad band between 15 and 40 degrees and was observed in all diffractograms of this batch of samples.

The diffractograms for BioRoot (Figure 5B), in contrast to the Bio‐C sample, detected only phase B of zirconium oxide besides tricalcium silicate. In addition, low evidences of calcium carbonate (calcite) and calcium phosphate (apatite) wereobserved. Amorphous material was not detected significantly exclusively for the sample dried for 24 h. All the other samples’ amorphous phase was identified.

## 4 Discussion

Concerns about canal moisture are prevalent among endodontists, particularly when using ready‐to‐use calcium silicate‐based sealers. These sealers rely on dentine moisture for proper setting, yet it remains unclear whether the water present in the dentine is sufficient to initiate the reaction with calcium silicate particles [9, 18]. There are few reports in the literature on the behaviour of these sealers under different canal drying protocols. Pelozo et al. (2023) [15] evaluated various drying techniques to determine the most effective for achieving satisfactory bond strength. Building on this, the present study aimed to assess the impact of clinical drying protocols on the setting and hydration of these materials.

To achieve this, we investigated how root canal dentine moisture influences the setting time of calcium silicate‐based sealers. This is the first study to compare setting times in canals with and without moisture using a novel methodology. Traditional ISO methods, which rely on plaster moulds or stainless‐steel rings, fail to accurately simulate dentine tubule moisture. In contrast, our approach utilised human teeth, divided by canal thirds, to better reflect clinical conditions. Additionally, a modified Gilmore needle, adapted for smaller canal diameters, was employed based on a methodology previously proposed by Silva et al. (2021) [17] to assess setting time in an in vivo‐like model.

For the Bio‐C sealer, the canal should be dried with paper points to ensure thorough drying without causing excessive desiccation, following the instructions of the manufacturer. Setting occurred more rapidly in slightly wet canals of the Bio‐C Sealer group compared to dry canals [17]. This outcome aligns with expectations, as tricalcium and dicalcium silicate react with water to form a hydrated calcium silicate matrix, facilitating the hardening of the material [19]. However, when root canals were not dried, this process did not occur. The setting time for dry canals, though delayed, was still ensured due to ambient humidity. Based on these findings, in clinical practice, keeping the canals slightly moist before filling will ensure a reduction in the setting time of these materials.

Regarding BioRoot, the presence of moisture in the canal did not significantly affect the setting reaction. Upon mixing the powder components with the liquid, the setting reaction initiated. The presence of water in the canals did not diminish the setting time for BioRoot. Furthermore, the presence of calcium chloride enhances the setting reaction of the material. Differently from the Bio‐C Sealer, BioRoot does not contain trace elements. It is composed of pure tricalcium silicate without the presence of aluminium, which has been demonstrated to reduce setting time [20].

This is the first study evaluating the hydration of sealers in root canals through FT‐IR and Raman spectroscopy. In Raman analyses, the calcite peak at 1150 cm^−1^ was successfully identified. Calcium carbonate results from the reaction between released calcium hydroxide and environmental carbon [8, 21], and it is identified in the set form of sealer after hydration [22]. Polyethylene glycol (PEG) peaks were detected exclusively in the spectra of fresh samples and those from the first day in the case of Bio‐C. The observed flattening of PEG peaks after 28 days suggests a potential reaction. Additional research is warranted to comprehensively investigate this phenomenon. In general, both fresh sealers and those assessed after 1 day exhibited no changes in Raman peaks.

The FT‐IR spectra of the Bio‐C Sealer in its fresh form demonstrated the presence of water, even in dry canals. Manufacturers claim that the vehicle of the Bio‐C Sealer does not contain water; however, the presence of the O‐H bond can be observed at 3400 cm^−1^. This could justify the setting of the sealer within the syringes in some batches. The FT‐IR spectra of the wet Bio‐C Sealer on the first day reveal prominent water peaks at 3400 cm^−1^ compared to the peaks of the dry Bio‐C Sealer during the same period, emphasising a higher concentration of water [23]. After 28 days, the water peaks become highly evident, attributed to environmental humidity. Both wet and dry BioRoot FT‐IR spectra display significant peaks in their fresh form, with more intense peaks compared to the Bio‐C. This is explained by the presence of water in the powder/liquid form, regardless of whether the canals are dry or moist [10]. Within a day, the peaks diminish as water is consumed during the setting process. However, in the wet group, the peaks are larger compared to those in the dry group due to a higher concentration of water. Similar to the Bio‐C group, after 28 days, the water peak increases, indicating water absorption from the environment.

The tricalcium and dicalcium silicate in sealers react with water to form a matrix of hydrated calcium silicate (C‐S‐H) and calcium hydroxide, which reacts in the presence of physiological fluids, producing hydroxyapatite [8, 24]. Previous studies have shown, through X‐ray diffraction, the presence of high peaks of calcium hydroxide in the reaction product of BioRoot powder and liquid after 28 days of cement disk immersion in PBS solution [25]. However, in the present study, this compound was not observed in BioRoot after 28 days in both dry and wet samples. The immersion of cement specimens in PBS solution may have influenced the formation of calcium hydroxide. There is a study in the literature, which also did not identify calcium hydroxide in BioRoot after immersion in water, corroborating the results of the present study [26]. Furthermore, other studies show small peaks of calcium hydroxide, contrasting with the findings of Khalil et al. 2016 [27, 28]. Apatite compounds were only identified in BioRoot sealer, which may be explained by the chemical interaction between dentine and the material [29]. After removing the sealer from the canal for XRD testing, portions of apatite might be included in the sample. It has been demonstrated that BioRoot only presents hydroxyapatite in PBS solutions and not in water [26]. The formation of mineral tags of BioRoot in the dentine [29] may justify the identification of this compound in the samples of the present study.

Analysing the changes in crystalline phases before and after hydration is crucial for comprehending the composition and setting process of these sealers [18]. This study aimed to qualitatively assess the crystalline phases in both powder and hydrated states of Bio‐C Sealer and BioRoot RCS sealers using XRD analysis, contributing to our broader understanding of dental cement behaviour. In Bio‐C Sealer, calcium hydroxide was identified at 18θ in wet samples through X‐ray diffraction, proving once again that this ready‐to‐use sealer does not set without moisture. Moisture is essential for forming calcium hydroxide, the hydration reaction product. Keeping the dentine slightly moist was crucial for this process, allowing the sealer to exert its bioactivity role.

In XRD analysis, calcium carbonate was only observed in BioRoot samples. Another study has also identified calcium carbonate in BioRoot through XRD in both PBS and distilled water samples [26]. Additionally, XRD revealed that Bio‐C Sealer has a higher proportion of radiopacifier (45.6%) than tricalcium silicate (36.5%), while BioRoot has a higher proportion of tricalcium silicate (53.7%) than radiopacifier (45.2%). This may indicate that the setting time of BioRoot is longer than that of Bio‐C Sealer.

Other previous studies have evaluated the drying protocol of calcium silicate‐based sealers through bond strength assessments [13, 14, 15]. However, none has investigated the setting time with slightly moist and dry canals. In all these studies, canals that remained slightly moist before filling demonstrated superior sealing results compared to both overly wet and extremely dry canals, with the moderate moisture level proving to be the optimal choice. The authors concluded that maintaining canals slightly moist before using hydrophilic sealers is advantageous and promotes better sealer penetration into dentinal tubules. In contrast, dry canals remove water from dentine, hindering sealer penetration.

In this study, gutta‐percha was not used because its presence could act as an additional variable, making it difficult to isolate the effect of dentinal moisture on the sealer. Furthermore, the evaluation of setting time required direct needle application to the material surface, which would not have been feasible with a gutta‐percha cone.

Some limitations of the present study must be acknowledged. The sealers were evaluated in the absence of gutta‐percha, which is routinely used in endodontic obturation, and only two calcium silicate‐based sealers were investigated, without comparison to other commercially available materials. These factors may restrict the direct extrapolation of the results to standard clinical procedures. In addition, while previous studies have reported that isopropyl alcohol may enhance the bond strength of resin‐based sealers to dentine [30], such findings relate to adhesion phenomena and not to the setting reaction, which was the focus of the present investigation. Bonding and setting are distinct properties and should not be directly compared. Therefore, further studies incorporating gutta‐percha, additional types of sealers and conditions more closely resembling clinical practice are needed to strengthen the clinical relevance of these findings. This work should be regarded as an exploratory study, providing a starting point and useful information for subsequent investigations on calcium silicate‐based sealers.

The findings of this work may contribute to the study of the hydration of hydraulic cements and the canal drying protocol that will be filled with calcium silicate‐based sealers. Further studies are needed to refine the filling technique and enhance understanding of the setting reaction and hydration of hydraulic sealers. One limitation of this study is the evaluation of hydration of the sealers in extracted teeth, which do not have the same physical and moist conditions as a natural tooth in the mouth.

## 5 Conclusions

The moisture of dentine was crucial for the setting of ready‐to‐use calcium silicate‐based sealers. Bio‐C Sealer was the only sealer that exhibited calcium hydroxide in the moist samples at 28 days.

## Author Contributions

Conceptualisation: Thiago Bessa, Bruno Guimarães and Marina Marciano. Methodology: Thiago Bessa, Ana Cristina, Isis Hinnebusch, Nilvan Alves, Gaspar Darin and Elda Xavier. Formal analysis: Marina Marciano, Bruno Guimarães, Mário Sinhoreti and Thiago Bessa. Investigation: Thiago Bessa, Ana Cristina and Isis Hinnebusch. Data curation: Nilvan and Gaspar Darin. Writing‐original draft preparation: Thiago Bessa and Bruno Guimarães. Writing‐review and editing: Marina Marciano, Bruno Guimarães and Mário Sinhoreti. All authors contributed significantly and are in agreement with the manuscript.

## Funding

This work was supported by Fundação de Amparo à Pesquisa do Estado de São Paulo (2021/11992‐0 & 2021/07920‐4). Coordenação de Aperfeiçoamento de Pessoal de Nível Superior (001).

## Ethics Statement

This study was approved by the Ethics Committee of the Piracicaba Dental School (Protocol number 54541321.4.0000.5418).

## Data Availability

The data that support the findings of this study are available on request from the corresponding author. The data are not publicly available due to privacy or ethical restrictions.
